# Hypoxia-associated markers in gastric carcinogenesis and HIF-2*α* in gastric and gastro-oesophageal cancer prognosis

**DOI:** 10.1038/sj.bjc.6604210

**Published:** 2008-02-19

**Authors:** E A Griffiths, S A Pritchard, S M McGrath, H R Valentine, P M Price, I M Welch, C M L West

**Affiliations:** 1 Academic Department of Radiation Oncology, School of Cancer & Imaging Sciences, The University of Manchester, Christie Hospital, Wilmslow Road, Withington, Manchester M20 4BX, UK; 2 Department of Gastrointestinal Surgery, South Manchester University Hospitals NHS Trust, Manchester M23 9LT, UK; 3 Department of Histopathology, South Manchester University Hospitals NHS Trust, Manchester M23 9LT, UK

**Keywords:** gastric cancer, gastro-oesophageal junction tumours, HIF-2*α*, hypoxia, carcinogenesis

## Abstract

The study investigated hypoxia-associated markers (HIF-2*α*, Epo, Epo-R, Glut-1 and VEGF) along with Ki-67 in a gastric carcinogenesis model, and the prognostic significance of hypoxia-inducible factor (HIF)-2*α* in surgically treated gastro-oesophageal cancer. Protein expression was examined using immunohistochemistry on formalin-fixed, paraffin-embedded biopsies of normal mucosa (*n*=20), *Helicobacter pylori*-associated gastritis (*n*=24), intestinal metaplasia (*n*=24), dysplasia (*n*=12) and intestinal (*n*=19) and diffuse (*n*=21) adenocarcinoma. Relationships between HIF-2*α* expression and prognosis were assessed in resection specimens from 177 patients with gastric and gastro-oesophageal junction adenocarcinoma. Expression of all markers increased with progression along the gastric carcinogenesis sequence (*P=*0.0001). Hypoxia-inducible factor-2*α* was expressed in 63% of 177 resection specimens and at a high level in 44%. The median overall survival in patients with HIF-2*α*-expressing tumours was 22 (95% CI 18−26) months, whereas those with HIF-2*α*-negative tumours had a median survival of 37 (95% CI 29−44) months (*P=*0.015). Hypoxia-inducible factor-2*α* had no independent prognostic significance in multivariate analysis. In view of the lack of independent prognostic significance, HIF-2*α* has no role as a routine prognostic indicator. However, the high expression of HIF-2*α* suggests that it may be of value as a potential therapeutic target.

Gastric and oesophageal cancers are among the most common malignancies worldwide and contribute significantly to global cancer mortality (Parkin *et al*, 2001). To improve the diagnosis and prognosis of gastric and gastro-oesophageal cancer, it is important to fully understand the molecular mechanisms underlying carcinogenesis. The well-described premalignant sequences of these tumour types make them ideal to study and explore potential molecular mechanisms of carcinogenesis by immunohistochemistry. Histologically, gastric cancers can be classified into two types: diffuse and intestinal. For the development of intestinal gastric cancer, a multistep process involving a progressive cascade of molecular and morphological changes has been proposed by Correa (2004). Diffuse tumours have no known premalignant precursor lesions. For both types of tumour, the carcinogenesis process is believed to be initiated by *Helicobacter pylori* infection and the risk of gastric cancer development has been related to *H. pylori* strain type, other environmental factors, host genetic factors and immune-related polymorphisms (Nardone *et al*, 2004).

Tumour hypoxia is a key factor driving the development of malignancy, and the master regulatory protein in the response of cells to changing oxygen levels is the hypoxia-inducible factor (HIF). Researchers have hypothesised that hypoxia plays a primary role in the carcinogenesis process. Studies have shown progressively increased HIF-1*α* expression in breast (Bos *et al*, 2001), skin (Costa *et al*, 2001) and cervical (Acs *et al*, 2003) cancer development. More recently, we have shown increased expression of HIF-1*α* in gastric cancer development (Griffiths *et al*, 2007b) and in adenocarcinoma *vs* dysplasia in the Barrett's oesophageal cancer sequence (Griffiths *et al*, 2007a). Hypoxia-inducible factor-1*α* is a key mediator of transcription and upregulates genes involved in a variety of processes: vascular endothelial growth factor (VEGF); erythropoietin (Epo) and its receptor (Epo-R), which regulates erythropoiesis by stimulating the growth and differentiation of red blood cell precursors (Yasuda *et al*, 2003; Ratcliffe, 2007). Erythropoietin and its receptor (Epo-R) are expressed in a number of cancers and are involved in breast (Acs *et al*, 2002), endometrial (Acs *et al*, 2004), melanoma (Kumar *et al*, 2005) and prostate (Feldman *et al*, 2006) tumorigenesis.

Recently, a number of proteins have been identified that are closely related to HIF-1*α* and control the transcription of hypoxia-regulated genes in a similar way (HIF-2*α* and HIF-3*α*) (Calzada and del Peso, 2007). A study in non-small cell lung cancer showed that HIF-2*α* expression was related to a poor outcome whereas HIF-1*α* expression was not (Giatromanolaki *et al*, 2001). Another study showed a predominant role of HIF-2*α* over HIF-1*α* in the regulation of the transcriptional response to hypoxia in renal cell carcinoma (Sowter *et al*, 2003). These findings raise the possibility of tissue-specific differences in the relative importance of HIF proteins in determining tumour progression and prognosis. We have previously shown that HIF-1*α* is involved in gastric carcinogenesis and invasive edge tumour expression is associated with an adverse prognosis (Griffiths *et al*, 2007b). Hypoxia-inducible factor-2*α* expression, however, has not yet been assessed as a prognostic marker in gastro-oesophageal cancer.

The hypothesis underlying the research is that hypoxia plays a role in the aetiology and prognosis of gastro-oesophageal cancer. The specific goals of the research were to investigate whether the expression of hypoxia-associated proteins increases along the gastric carcinogenic sequence. Three hypoxia-associated markers were selected that have not been assessed in gastric carcinogenesis: HIF-2*α*, Epo and Epo-R. Although studied previously, VEGF and Glut-1 were also studied. The widely investigated Ki-67 was included as a comparator. The expression of the proteins was assessed using immunohistochemistry in paraffin-embedded material representing the gastric carcinogenesis sequence. A further aim was to assess the prognostic value of HIF-2*α* expression in surgically treated gastric and gastro-oesophageal cancer patients.

## PATIENTS AND METHODS

### Tissue specimens

The study was approved by the South Manchester Ethics Committee. Tissues were obtained from the Department of Histopathology, South Manchester University Hospitals NHS Trust. Formalin-fixed endoscopic gastric biopsy samples obtained were of normal gastric mucosa (*n*=20), *H. pylori*-associated gastritis (*n*=20), intestinal metaplasia (*n*=20), epithelial dysplasia (*n*=12) and intestinal (*n*=19) and diffuse (*n*=21) gastric adenocarcinoma. Four of the biopsies had both *H. pylori*-infected mucosa and intestinal metaplasia. Haematoxylin and eosin slides were reassessed by a consultant pathologist (SP) to ensure correct classification. All cases of *H. pylori*-associated gastritis showed significant numbers of organisms. The epithelial dysplasia group was classified as low- (*n*=6) or high- (*n*=6) grade. Intestinal metaplasia was present in 6 of the 12 dysplasia biopsies.

### Surgically treated patients

A retrospectively compiled database was established of 251 consecutive patients with primary gastric and gastro-oesophageal junction tumours who underwent surgery at the South Manchester University Hospitals NHS Trust between 1995 and 2004. The Siewert classification was used to classify gastro-oesophageal junction tumours (Siewert and Stein, 1998). Patients who had either Siewert type I gastro-oesophageal tumours (*n*=22), neo-adjuvant therapy (*n*=31), emergency surgery (*n*=1), completion gastrectomy (*n*=6) or died after surgery (*n*=25) were excluded from the study. The study group therefore comprised 177 patients (125 men) with a median age of 68 (range 49–85) years. There were 76 Siewert type II, 21 type III gastro-oesophageal junction tumours and 80 noncardia gastric cancers. Patients underwent either partial or subtotal gastrectomies (*n*=45), total gastrectomy (*n*=44), proximal gastrectomy (*n*=4) or oesophago-gastrectomy (*n*=84). Selected patients underwent additional surgical resection of the spleen (*n*=21) and spleen with distal pancreas (*n*=5). One hundred and thirteen patients (64%) underwent a potentially curative resection (R0). Fifty-four patients (31%) had residual microscopic disease (R1 resection) and 10 patients (6%) had residual macroscopic disease (R2 resection). After surgery, patients were followed in the surgical outpatient clinic. Hospital notes of the patients were reviewed and, if necessary, the local cancer registry or patient's general practitioner was contacted to complete case follow-up.

### Immunohistochemistry

Antigen retrieval was carried out by microwaving for 25 min in either 10 mM sodium citrate (pH 6.0) or 0.05 M Tris-HCl (Sigma-Aldrich Ltd., Poole, UK)/1 mM EDTA (Sigma) (pH 8.5 or 9.0) buffer solution. After quenching endogenous peroxidase, nonspecific binding was blocked using 10% casein solution (Vector Laboratories Ltd., Peterborough, UK). The primary antibody was applied and the sections incubated as described elsewhere (Griffiths *et al*, 2007a). Mouse or rabbit EnVisionPlus System (Dako, UK) was used for antigen detection. Identical concentrations of immunoglobulin IgG1 (Dako Ltd., Ely, UK) from the same species were used as negative controls. Positive and negative (or low) tissue controls from gastric, cervical or head and neck cancer with known staining characteristics were used in each batch. Batch-to-batch variation was assessed by running sections showing high and low protein expression with each batch. Antibodies were visualised with 3,3′-diaminobenzidine (Dako) and the sections lightly counterstained with haematoxylin, dehydrated and coverslipped.

### Scoring

Scoring was performed in a double-blind manner by two investigators (SAP, SMG). Any disagreement was resolved by discussion to obtain final scores. Markers (HIF-2*α*, VEGF, EPO, EPO-R, Glut-1, and Ki-67) in the carcinogenesis study were scored using the same scoring system. A score (0–300) was calculated for each marker by multiplying intensity (none 0, weak 1, moderate 2, strong 3) with percentage of expression (range 0–100). For the prognostic study, tumour nuclear HIF-1*α* and HIF-2*α* staining was scored as follows: 0, no staining; 1, <2% staining; 2, 2–10% staining; 3, 11–29% staining; and 4, >30% staining. Hypoxia-inducible factor-1*α* scores were obtained from our previous research (Griffiths *et al*, 2007b).

### Statistics

Data were analysed using SPSS version 11.5. The Spearman's rank test was used to investigate relationships between variables. Differences in expression levels in the carcinogenesis studies were assessed using Mann–Whitney and Kruskal–Wallis tests. The Jonckheere–Terpstra test was used to identify ordered differences in marker expression. With this test, the null hypothesis is that distributions do not differ across ordered categories. The χ^2^ test was used to correlate tumour HIF-2*α* expression with clinico-pathological characteristics.

Survival time was measured as the time from the date of surgery until death or last follow-up appointment. Overall and cancer-specific survival were used as end points. At the time of analysis, 51 patients were alive with a median follow-up of 48 (range 13–118) months and 107 had died of disease with a median time to death of 14 (range 2–74) months. There were 16 intercurrent deaths from other causes. Univariate survival analyses were illustrated using the Kaplan–Meier method. Factors were compared using the Cox proportional hazards model and log-rank tests. Multivariate survival analysis was performed on factors that achieved statistical significance in univariate analysis, using the Cox proportional hazards model to identify independent predictors of survival. All statistical tests were two-sided at the 0.05 significance level. As adjusting statistical significance depending on the number of tests performed can create problems (Perneger, 1998), no allowance was made for multiple testing.

## RESULTS

### Staining

**Figure 1:**
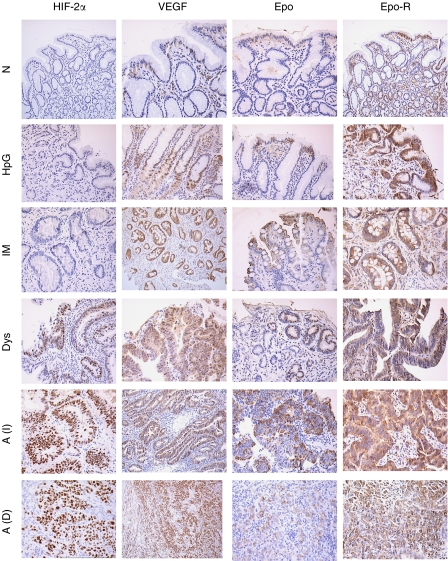
Representative photomicrographs of HIF-2*α*, VEGF, Epo, Epo-R immunohistochemistry in the gastric cancer progression sequence. A(I)=intestinal adenocarcinoma; A(D)=diffuse adenocarcinoma; Dys=dysplasia; Epo=erythropoietin; Epo-R=erythropoietin receptor; HIF-2*α=*hypoxia-inducible factor-2*α*; HpG=*H. pylori* gastritis; IM=intestinal metaplasia; *N*=normal mucosa; VEGF=vascular endothelial growth factor.

Figure 1 shows photomicrographs of HIF-2*α*, VEGF, Epo and Epo-R staining in the gastric cancer progression sequence. Only nuclear HIF-2*α* staining was scored, however, occasional cytoplasmic staining was seen. Hypoxia-inducible factor-2*α* was not expressed in normal tissue. Low levels of expression were found in *H. pylori* gastritis and intestinal metaplasia biopsies. Vascular endothelial growth factor staining was cytoplasmic. Normal gastric mucosa showed weak staining mainly in the deep portions of the crypts that tended to be basal in location, in cytoplasm around the nucleus. Areas of intestinal metaplasia showed increased staining compared with normal mucosa and adjacent nonmetaplastic mucosa within the same biopsy. Erythropoietin staining was cytoplasmic and predominantly focal in nature; Epo-R was expressed in cytoplasm and membrane. Inflammatory cells acted as an internal positive control. Glut-1 staining was cytoplasmic and/or membranous, and detected only in invasive cancer biopsies. In normal gastric mucosa, nuclear Ki-67 staining was present mainly in the cells in the neck region of the gastric pits. In neoplastic tissue, this normal staining pattern was lost and more diffuse expression seen. In all cases along the gastric carcinogenesis sequence, when specialised gastric body-type cells (chief and parietal cells) were present in the biopsy, they showed intense staining for VEGF and Epo-R in a uniform fashion. As the staining was identical in all cases these areas were not scored.

### Expression of markers along the gastric carcinogenesis sequence

**Figure 2:**
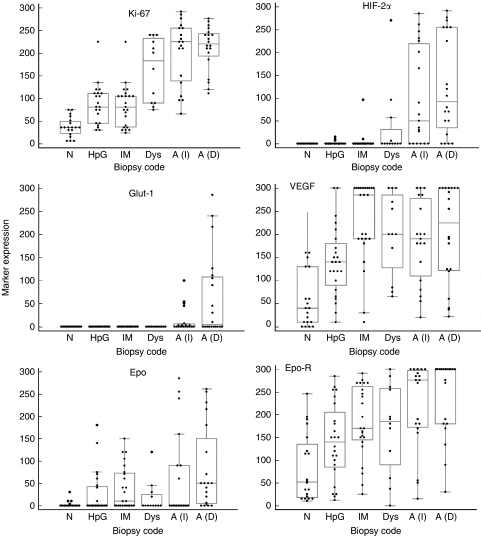
Box and whisker plots of each immunohistochemical marker in the gastric carcinogenesis sequence. The box represents 25–75 quartiles with a median line. The whiskers extend to minimum and maximum values, but exclude outlying and far-out values. A(I)=intestinal adenocarcinoma; A(D)=diffuse adenocarcinoma; Dys=dysplasia; Epo=erythropoietin; Epo-R=erythropoietin receptor; HIF-2*α=*hypoxia-inducible factor-2*α*; HpG=*H. pylori* gastritis; IM=intestinal metaplasia; *N*=normal mucosa; VEGF=vascular endothelial growth factor.

**Figure 3:**
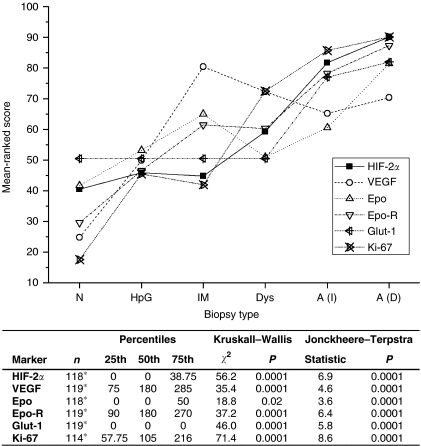
The mean rank score (Kruskall–Wallis test) of each marker in relation to the gastric carcinogenesis sequence. Immunohistochemical score was calculated from percentage (0–100) multiplied by intensity (0–3) of expression for each marker studied. ^*^Insufficient biopsy tissue for marker scoring in some sections. The Kruskall–Wallis and Jonckheere–Terpstra test results for each immunohistochemical marker studied in the gastric carcinogenesis sequence. A(I)=intestinal adenocarcinoma; A(D)=diffuse adenocarcinoma; Dys=dysplasia; Epo=erythropoietin; Epo-R=erythropoietin receptor; HIF-2*α=*hypoxia-inducible factor-2*α*; HpG=*H. pylori* gastritis; IM=intestinal metaplasia; *N*=normal mucosa; VEGF=vascular endothelial growth factor.

Interobserver agreement for marker scores was highly statistically significant and consistent for all the markers studied (*P*<0.0001 for all). With the exception of Glut-1 and VEGF, there were statistically significant correlations between the expression levels of the various markers. For example, HIF-2*α* expression correlated with VEGF (*r*=0.20, *P=*0.03), Epo (*r*=0.34, *P*<0.001), Epo-R (*r*=0.46, *P*<0.001), Glut-1 (*r*=0.38, *P*<0.001), Ki-67 (*r*=0.59, *P=*0.59, *P*<0.001) and also HIF-1*α* (*r*=0.34, *P*<0.001). Box and whisker plots (including individual data points) are shown in Figure 2 and mean rank scores (from the Kruskall–Wallis test statistics) are plotted in Figure 3. There was a statistically significant increase in the expression of all markers along the progression sequence to adenocarcinoma.

### Expression of HIF-2*α* in surgically resected specimens

Tumours tended to show diffuse staining for HIF-2*α* in almost all nuclei or negative staining (Figure 1). There was no obvious association with inflammation, ulceration or infiltrative edge and location of HIF-2*α* positive staining. In nine slides, cytoplasmic staining was present; this was not scored. Focal staining was identified in some inflammatory cells that acted as an internal positive control. Five sections (2.8%) had insufficient tissue for HIF-2*α* scoring. In 66 sections (37.3%), no HIF-2*α* immunostaining was observed. Positive nuclear staining was as follows: <2% staining in 9 sections (5.1%), 2–10% staining in 13 sections (7.3%), 11–30% staining in 6 sections (3.4%) and >30% staining in 78 sections (44.1%). All negative controls showed no immunoreactivity. No statistically significant relationships were found between the expression of HIF-1*α* (Griffiths *et al*, 2007b) and HIF-2*α* (*P=*0.31).

### HIF-2*α* expression and clinicopathological features

For correlation with various clinicopathological features, HIF-2*α* expression was categorised as negative (score 0) and positive (scores 1/2/3/4). The distribution of patients according to tumour HIF-2*α* expression is shown in Table 1. Hypoxia-inducible factor-2*α*-positive tumours were more likely to be diffuse (*P=*0.025). There was also a trend for HIF-2*α* tumours to have a more advanced T stage (*P=*0.058). No statistically significant correlations were found between HIF-2*α* and differentiation, N stage, M stage, overall TNM stage or R classification.

### HIF-2*α* expression and patient survival

**Figure 4:**
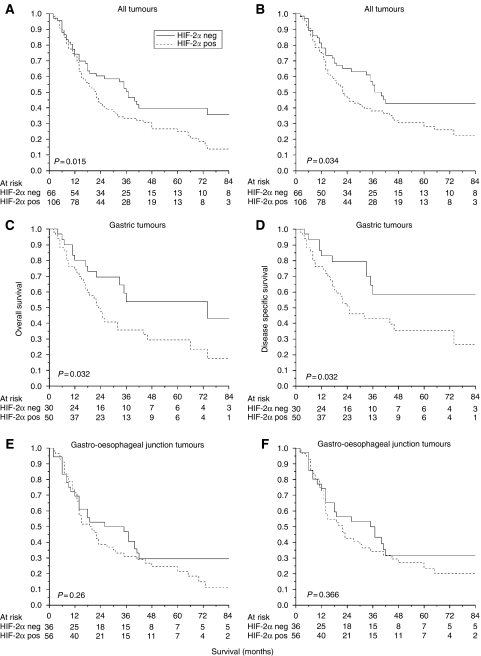
Hypoxia-inducible factor-2*α* expression and patient outcome in 172 tumours including noncardia gastric cancers (*n*=80) and gastro-oesophageal junction tumours (*n*=92). The first column shows HIF-2*α*-negative (score 0) *vs* positive (scores 1/2/3/4) expression and overall survival. For all tumours (**A**), gastric cancers (**C**) and gastro-oesophageal junction tumours (**E**). Second column shows disease-specific survival. For all tumours (**B**), gastric tumours (**D**) and gastro-oesophageal junction tumours (**F**). HIF*=*hypoxia-inducible factor.

Tumour HIF-2*α* expression was a statistically significant adverse prognostic factor (Figure 4 and Table 2). The median overall survival for patients with tumour HIF-2*α* expression was 22 (95% CI 18−26) months, whereas HIF-2*α*-negative patients had a median survival of 37 (95% CI 29−44) months (*P=*0.015). Hypoxia-inducible factor-2*α* expression was more prognostic for gastric (*P=*0.032) than gastro-oesophageal (*P=*0.26) tumours (Figure 4). Other significant factors in univariate survival analyses were tumour differentiation, T stage, N stage, overall TNM stage and R classification (Table 2).

**Figure 5:**
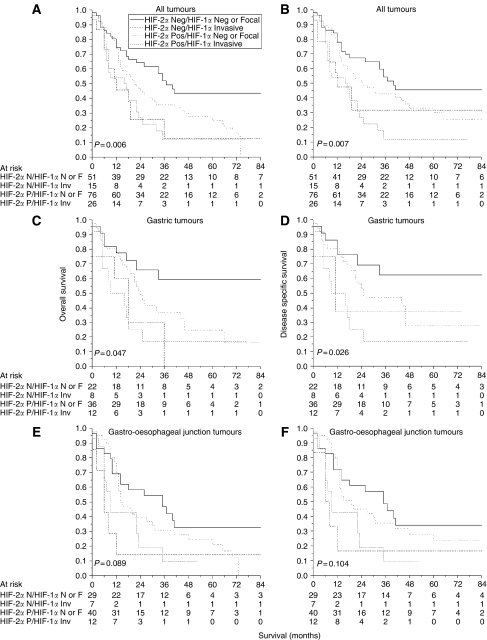
Combined HIF-1*α* and HIF-2*α* expression and patient outcome in 168 tumours including noncardia gastric cancers (*n*=78) and gastro-oesophageal junction tumours (*n*=90). The first column shows overall survival (**A**, **C**, **E**) and the second column shows disease-specific survival (**B**, **D**, **F**). HIF*=*hypoxia-inducible factor.

The combined effect of tumour HIF-1*α* (Griffiths *et al*, 2007b) and HIF-2*α* expression was analysed in relation to patient outcome. Patients with invasive edge HIF-1*α*- and HIF-2*α*- expressing tumours had a poorer overall survival than those with HIF-1*α*-negative/focally positive and HIF-2*α*-negative cancers (*P=*0.006). The median overall survival times were 17 (95% CI 4−30) and 40 (95% CI 32−42) months, respectively. Corresponding figures for cancer-specific survival were 41 and 17 (95% CI 4−30) months (*P=*0.007). As for HIF-1*α* (Griffiths *et al*, 2007b) and HIF-2*α* (Figure 4) alone, the combined adverse effect of invasive edge HIF-1*α* and HIF-2*α* expression was greater for gastric than for gastro-oesophageal junction tumours for both disease-specific (*P=*0.026 *vs* 0.104) and overall (*P=*0.047 *vs* 0.089) survival (Figure 5). For example, patients with HIF-1*α*-negative/focally positive and HIF-2*α*-negative gastric cancers had an average disease-free survival of 81 (95% CI 59−103) months compared with 25 (95% CI 11−39) months for those with invasive edge HIF-1*α*- and HIF-2*α*-positive tumours. All analyses were repeated stratifying patients by 0/1 *vs* 2/3/4 scores. Although qualitatively similar results were seen, they were not statistically significant.

All factors that achieved statistical significance (*P*<0.05) in the univariate analysis were entered into a multivariate analysis. Neither HIF-2*α* expression nor HIF-1*α*/HIF-2*α* combined had any independent prognostic significance. Only tumour differentiation, overall TNM stage and R classification retained significance in multivariate analysis.

## DISCUSSION

All markers studied showed a statistically significant increase in expression with progression from *H. pylori*-associated gastritis, intestinal metaplasia, dysplasia to adenocarcinoma (*P=*0.0001). It must be emphasised, however, that this model does not apply to gastro-oesophageal junction tumours. Gastro-oesophageal adenocarcinomas (Siewert type I and II tumours) arise via a similar sequence of histopathological events; however, the initiating, promoting and molecular factors are different to gastric cancer carcinogenesis. Indeed, *H. pylori* appears to exert a protective role in these types of tumours (Chow *et al*, 1998). We have previously assessed these immunohistochemical markers in a similar model of oesophageal carcinogenesis (Griffiths *et al*, 2007a).

Previous studies have shown the importance of Ki-67 expression and proliferation in the progression of gastric cancer. For example, Leung *et al* (2001) showed that proliferation, as measured by Ki-67 expression, was significantly increased in both *H. pylori*-infected and intestinal metaplasia biopsy material. Interestingly, they also found that the apoptotic to proliferation index ratio was significantly reduced, favouring cellular DNA-damage accumulation and neoplastic progression. Similarly, the role of VEGF and angiogenesis in the gastric carcinogenesis sequence has been previously reported. We found similar results to that of Feng *et al* (2002), who assessed VEGF expressed by immunohistochemistry. Our finding that Glut-1 protein is not found in normal gastric mucosa but only expressed in gastric carcinoma also confirms the results of others (Younes *et al*, 1996; Kim *et al*, 2000; Kawamura *et al*, 2001). For example, one study reported no expression of Glut-1 in normal gastric mucosa, intestinal metaplasia and tubular adenomas but 30% of 617 gastric carcinomas was positive for the protein (Kawamura *et al*, 2001).

Erythropoietin and Epo-R have been shown to be expressed in a number of cancers and involved in breast (Acs *et al*, 2002), endometrial (Acs *et al*, 2004), melanoma (Kumar *et al*, 2005), prostate (Feldman *et al*, 2006) and Barrett's oesophageal (Griffiths *et al*, 2007a) carcinogenesis. There appear to be no other studies that have assessed these proteins in gastric carcinogenesis. Both Epo and Epo-R were expressed in varying degrees in all biopsy types, but Epo-R was expressed more abundantly. The expression of both proteins increased from normal tissue through to invasive cancer. Endogenous Epo is a glycoprotein produced primarily in the adult kidney in response to hypoxia. Its main function is to regulate erythropoiesis by stimulating growth, inhibiting apoptosis and inducing differentiation of red blood cell precursors (Yasuda *et al*, 2003).

Hypoxia-inducible factor-2*α* expression occurred principally as a late phenomenon. However, there was evidence of expression in a minority of the earlier premalignant biopsies of *H. pylori* gastritis and intestinal metaplasia. Hypoxia-inducible factor-2*α* expression has not been studied in models of tumour carcinogenesis except in our work in the Barrett's metaplasia–dysplasia–adenocarcinoma sequence, where we showed that HIF-2*α* was expressed late in the sequence and was seen only in dysplasia and adenocarcinoma (Griffiths *et al*, 2007a). Recently, the overexpression of HIF-2*α* in rat gliomas was shown to have counteracting properties, in which angiogenesis was induced but reduced growth was also found due to increased tumour-cell apoptosis (Acker *et al*, 2005). Hypoxia-inducible factor-2*α* expression has also been suggested to be critical in the carcinogenesis of renal cortical tumours (Kim *et al*, 2006).

Hypoxia-inducible factor-2*α* accumulates in the presence of hypoxia, forms a heterodimer with HIF-1*β* and binds to hypoxia-responsive elements. Hypoxia-inducible factor-2*α* has been shown to regulate a number of the same hypoxia-inducible genes as HIF-1*α* (Hu *et al*, 2003). However, it is now known that the various hypoxia-inducible genes vary in their sensitivity to HIF-1*α* and HIF-2*α* (Wang *et al*, 2005) and therefore different downstream pathways can be preferentially activated (Hu *et al*, 2003; Sowter *et al*, 2003). The classical ‘hypoxic’ expression of HIF-2*α* in tumour sections was not found in our study as there was no association with necrosis or distance from blood vessels. This may suggest nonhypoxic activation. However, unlike HIF-1*α*, the nonhypoxic activation of HIF-2*α* has not been confirmed. A study in breast adenocarcinoma showed a strong correlation between HIF-2*α* and c-erbB-2 and suggested that this was due to oncogenic rather than hypoxic activation (Giatromanolaki *et al*, 2006). To clarify these issues, measurements of the oxygenation status of gastric cancers are required (Griffiths *et al*, 2005). Oxygen electrodes have proved useful in a number of tumour types (Nordsmark *et al*, 2003), but have limited use in gastric cancer because of poor accessibility. Other approaches such as the immunohistochemical expression of hypoxia-specific markers such as pimonidazole (Nordsmark *et al*, 2003) and noninvasive imaging (Koch and Evans, 2003) are being developed and could be carried out in patients with gastric cancer.

Few studies have assessed both HIF-1*α* and HIF-2*α* in relation to patient outcome. Yoshimura *et al* (2004) examined 87 surgically treated patients with colorectal cancer and found that HIF-2*α* but not HIF-1*α* expression predicted prognosis in univariate analysis. Other studies in nonsmall cell lung cancer and malignant melanomas showed that HIF-2*α* expression was related to a poor outcome when HIF-1*α* was not (Giatromanolaki *et al*, 2001, 2003). These studies confirm the likely tissue-specific differences in the relative importance of HIF proteins in determining tumour progression and prognosis.

The expression of HIF-2*α* was significant in univariate analysis; however, it was not an independent predictor of prognosis. In view of the lack of independent prognostic significance, HIF-2*α* is unlikely to impact on clinical management. However, the high expression of HIF-2*α* in gastric and gastro-oesophageal junction tumours suggests that it may be of value as a potential therapeutic target.

## Figures and Tables

**Table 1:** The distribution of patient characteristics according to tumour expression of HIF-2*α* (*n*=172)

**Factor**	**HIF-2*α* negative**	**HIF-2*α* positive**	***P*** ^*^
*Differentiation*			
Well	8	10	
Mod	24	41	
Poor	34	55	0.84
			
*Lauren type*			
Diffuse	27	62	
Intestinal	39	44	0.025
			
*T stage*			
T *in-situ*	0	3	
T1	7	9	
T2	27	26	
T3	32	64	
T4	0	4	0.058
			
*N stage*			
N0	20	29	
N1	37	62	
N2	7	13	
N3	2	2	0.921
			
*M stage*			
M0	65	103	
M1	1	3	0.58
			
*Overall TNM stage*			
0	0	3	
I	14	15	
II	23	31	
III	27	50	
IV	2	7	0.31
			
*R class*			
R0	46	64	
R1	19	33	
R2	1	9	0.13

HIF*=*hypoxia-inducible factor.

^*^χ^2^ *P*-value.

**Table 2:** Univariate survival analyses

	**Overall survival**			**Disease-specific survival**		
**Parameter**	**HR**	**95% CI**	*P* ^*^	**HR**	**95% CI**	***P*** ^*^
*HIF-1α*						
0	1	—	—	1	—	
1/2/3/4	1.1	0.8–1.4	0.62	1.0	0.7–1.5	0.82
*HIF-1α*						
Negative	1	—	—	1	—	—
Focal	0.9	0.5–1.3	0.49	0.7	0.5–1.2	0.26
Invasive edge	1.6	1.0–2.4	0.042	1.6	1.0–2.5	0.047
						
*HIF-2α*						
0	1.0	—	—	1.0	—	—
1/2/3/4	1.6	1.1–2.4	0.018	1.6	1.0–2.4	0.038
						
*HIF-1α/HIF-2α*						
HIF-1*α* neg/HIF-2*α* neg	1.0	—	—	1.0	—	—
HIF-1*α* pos/HIF-2*α* neg	1.5	0.8–2.9	0.224	1.1	0.6–2.2	0.76
HIF-1*α* neg/HIF-2*α* pos	2.0	1.2–3.6	0.014	1.6	0.9–3.0	0.095
HIF-1*α* pos/HIF-2*α* pos	1.8	1.1–3.3	0.036	1.6	0.9–2.9	0.12
						
*HIF-1α/HIF-2α*						
HIF-2*α* neg/HIF-1*α* neg/focal	1.0	—	—	1.0	—	—
HIF-2*α* neg/HIF-1*α* inv	2.1	1.0–4.3	0.37	1.7	0.7–3.7	0.22
HIF-2*α* pos/HIF-1*α* neg/focal	1.8	1.1–2.8	0.16	1.5	0.9–2.5	0.10
HIF-2*α* pos/HIF-1*α* inv	2.6	1.5–4.6	0.001	2.7	1.5–4.8	0.001
						
*Diff*						
Well	1.0	—	—	1.0	—	—
Mod	2.9	1.4–6.2	0.005	3.4	1.3–8.5	0.011
Poor	3.7	1.8–7.8	0.001	5.3	2.1–13.3	0.001
						
*Lauren type*						
Intestinal	1.0	—	—	1.0	—	—
Diffuse	1.4	1.0–2.0	0.052	1.8	1.2–2.6	0.003
						
*Location*						
Non-GOJ	1.0	—	—	1.0	—	—
GOJ	1.4	1.0–2.0	0.083	1.5	1.0–2.2	0.059
						
*T stage*						
T0/1	1.0	—	—	1.0	—	—
T2	2.6	1.0–6.7	0.052	5.2	1.2–22.0	0.023
T3	4.8	1.9–12.0	0.001	9.6	2.3–39.0	0.002
T4	16.8	4.4–64.2	0.0001	37.5	6.8–207.6	0.0001
						
*N stage*						
N0	1.0	—	—	1.0	—	—
N1	2.0	1.3–3.0	0.003	2.5	1.5–4.1	0.001
N2	3.5	1.9–6.4	0.0001	4.8	2.5–9.2	0.0001
N3	4.2	1.5–12.0	0.008	5.7	1.9–16.9	0.002
						
*M stage*						
M0	1.0	—	—	1	—	—
M1	2.6	1.0–7.1	0.062	2.9	1.1–7.9	0.037
						
*Overall TNM stage*						
0/1	1.0	—	—	1.0	—	—
2	1.4	0.8–2.6	0.25	1.8	0.9–3.6	0.12
3	3.3	1.9–5.9	0.0001	4.5	2.3–8.8	0.0001
4	7.6	3.3–17.5	0.0001	10.9	4.4–27.1	0.0001
						
*R class*						
R0	1.0	—	—	1.0	—	—
R1	2.3	1.6–3.3	0.0001	2.7	1.8–4.0	0.0001
R2	5.8	2.9–11.6	0.0001	7.2	3.6–14.5	0.0001

CI=confidence interval; diff=differentiation; GEJ=gastro-oesophageal junction; HIF*=*hypoxia-inducible factor; HR=hazard ratio.

^*^P-value from a univariate Cox-proportional hazards model. HIF-1*α* results obtained from Griffiths *et al* (2007b).
